# Anatomy, Physiology, and Pathology

**Published:** 1837-04

**Authors:** 


### PART THIRD.

### Selections from the Foreign Journals.

---

### ANATOMY, PHYSIOLOGY, PATHOLOGY.

### *On Internal Strangulations of the Intestines.* By Dr. Charles Rokitansky, Professor of Pathology in the University of Vienna.

[We call the particular attention of our readers—medical and surgical,—to the following memoir, which we think will be found to possess peculiar interest. It relates to a class of cases which are the source of much distress to the practitioner, and often of much misconception as to their precise character.]

Dr. Rokitansky divides internal strangulations of the intestines into the three following species:

First Species. *The narrowing or complete obliteration of the canal of a piece of intestine, resulting from the pressure exerted on it at one or more spots, by a smaller or larger portion of intestine or its mesentery, so as to compress it against the opposite wall of the abdomen.*

It is plain that, in cases of this description, the pressure must be chiefly made in the direction of the posterior unyielding wall of the abdomen, and that any effects produced by pressure in the opposite direction must, for obvious reasons, be only very slight and transient. Another observation, the truth of which experience confirms, is, that it is the small intestine or its mesentery which exercises the pressure, and that the large intestine is generally the part that suffers; or, in other words, that the more moveable is most likely to fall upon and compress the more fixed portion of the intestine. Four cases of this species of strangulation are given.

Case i. The following phenomena were observed in the abdominal cavity of the body of a sugar-baker, aged forty-eight, who died in the General Hospital at Vienna, in 1827. On opening the abdomen, the upper curvature of the sigmoid flexure of the colon was found compressed by the mesentery of the small intestine, which lay in the cavity of the pelvis. The mesentery formed in this situation a roundish peduncle, about three inches long, and an inch and a half in thickness, from which the whole convoluted intestine hung. This string or peduncle, made tense by the weight of the intestine attached to it, lay over the aforesaid portion of the sigmoid flexure, and presented at certain points a tendinous appearance. The colon and a portion of the small intestine were considerably distended.

Case ii. A woman, aged eighty-seven, died in the General Hospital at Vienna, in December, 1834, with symptoms of strangulated hernia. On opening the abdomen, the principal part of the small intestine was found lying in the cavity of the pelvis; five-sixths of this intestine hung from its mesentery, as from a peduncle, over the lumbar vertebræ, into the pelvic cavity. The convolutions were glued together and to the walls of the pelvis by lymph. A small inguinal hernia existed on the right side, containing a portion of omentum. The upper sixth of the small intestine, on issuing from beneath the transverse mesocolon, formed a circular loop, and then, passing under the peduncle above mentioned, towards the right lateral region of the abdomen, made here another circular turn, ran behind the posterior surface of the peduncle, which was flattened by the spinal column, and then descended into the pelvis. In this situation, the upper sixth of the small intestine had suffered a remarkable degree of compression from the peduncle of mesentery and the convolutions attached to it. The compressed portion of intestine was moderately congested, but was considerably distended with fluid feces. The remaining portion of the small intestine was of a dark red colour, congested, inflamed, and friable. The large intestine was pale and contracted.

Case iii. A man, aged seventy-six, died in the General Hospital at Vienna, in August 1828. For a long time before death, he had laboured under marasmus, and complained of frequently recurring abdominal affections, accompanied by spasms of the stomach, tympanitic swelling of the belly, borborygmi, and obstinate costiveness. In addition to some morbid alterations in the liver and stomach, the following phenomena were observed on dissection. The sigmoid flexure of the colon, enlarged in its diameter to the extent of five inches; had passed upwards across the umbilical region into the right hypochondrium, and pushed the transverse colon and omentum towards the left side. The space below the umbilicus, even on this side, and the cavity of the pelvis, were filled with the convolutions of the small intestine, which hung over the portion of the sigmoid flexure that lay across the vertebral column, and compressed it by means of its mesentery. Above this spot, the sigmoid flexure was of a morbid red colour, its coats above a line in thickness, and its mucous lining, as well as that of the rectum near the sphincter ani, congested. The lower part of the ileum was connected to the ascending portion of the sigmoid flexure by a strong band of false membrane.

Case iv. A baker, aged eighty-seven, who had laboured under a very old and large hernia, was brought into the General Hospital at Vienna, on the 18th of November, 1828, with symptoms of strangulation of the intestine, and died shortly after admission. On opening the abdomen, the following circumstances were noticed. The intestines were excessively distended, and had compressed the liver and stomach, so as to alter their position very considerably. The small intestine was covered with purplish spots, greatly distended, its tissues very much congested, and its peritoneal coat thickened by patches of effused lymph. The cæcum and ascending colon, whose coats exhibited the same alterations, were distended with air and feces to a diameter of four inches. The lower part of the small intestine lay in the hernial sac, while the remaining portion, several convolutions of which were united by false membranes, was, together with a very long mesentery, thrown upwards into the right hypochondrium, across the right flexure of the colon, and exercised such a degree of pressure on the latter, by means of the weight of its contents (which amounted to four or five pounds), that air could not be forced through the compressed part of the colon. The transverse and descending colon, and the rectum, were pale, and reduced in their diameter.

From the foregoing cases it appears, that this species of strangulation occurs most frequently in persons considerably advanced in life, and that the tendency to its formation is caused by length and laxity of the mesentery, fecal accumulations in the bowels, large and old herniæ, and adhesions of the convolutions of the intestines to each other and to the mesentery. It is generally slow in its progress, and may terminate in complete strangulation, in cases where the portion of intestine immediately above the compressed spot is so loose and distensible as to admit of being folded over the strictured part.

Second Species. This may be termed *Rotatory*, as it consists in *the rotation of one part round an axis*, formed by some other part: it includes three sub-species:—1st, *the rotation of a portion of intestine round its own axis*; 2d, *round an axis formed of the mesentery*; 3d, *where a portion of intestine forms the axis round which another larger portion with its mesentery turns, so as to touch the periphery of the axis at every point*.

Of this form of strangulation, which is only a more complete development of the first species, the following cases are given:

Case i. A man, aged fifty-four, died in the General Hospital at Vienna, in December, 1834, with symptoms of strangulated hernia. In addition to extensive inflammation and adhesions of the peritoneum, the following circumstances were observed:—The small intestine, (with the exception of a portion contained in the hernial sac), the cæcum, and the ascending colon were enlarged to four times their normal diameter. The small intestine lay in folds closely pressed together, while the ascending colon with the cæcum, distended with air and feces, projected far beyond all the rest of the intestines, and was twisted inwards and around its own axis, so as to lie almost parallel to the transverse colon, and form with it such an extremely acute angle, as to obstruct the further passage of the contents of the intestine. The remaining portion of the colon and the rectum were pale and narrowed in their caliber.

CASE II. A woman, aged seventy-one, was admitted into the General Hospital in December, 1830, with symptoms of strangulated hernia, which was reduced by the taxis soon after her arrival. Ten days afterwards she died. The hernia had occurred in her fiftieth year, and had never increased beyond the size of a pigeon's egg, but she was during the latter part of her life subject to repeated attacks of obstinate costiveness, colicky pains, and inclination to vomit. On opening the body, organic derangements of the solid viscera of the abdomen were observed, with traces of peritoneal inflammation. The mesentery, which was folded and rolled up at the bottom, formed a spindle-shaped axis, four inches and a half in length, and an inch and a half in thickness, round which the small intestine was twisted. From the termination of the duodenum, the jejunum descended towards the right iliac fossa, and twisted itself in front of the spine round the axis before mentioned, which, by means of the convolutions attached to it, compressed the portion of intestine which passed under it. From this point the jejunum made two turns more round the axis of mesentery. The ileum then made three revolutions, and passing towards the right iliac fossa, covered for the most part by the windings of the jejunum, went to join the cæcum. The last portion of the ileum was greatly compressed, scarcely a finger's breadth in diameter, its coats discoloured, soft, and friable; about two inches from the valve of the cæcum, it exhibited a perforation capable of admitting a hazel nut.

CASE III. A woman, aged seventy-two, who had suffered for many years from costiveness, pain, and distention of the abdomen, was admitted into the General Hospital on the 25th of June, 1833, with symptoms of fever, epigastric tenderness, tympanitis, and nausea. On the following day she had feculent vomiting, which was frequently repeated, and she died on the 28th. On opening the abdomen, traces of peritoneal inflammation were found, together with numerous adhesions. The mesentery was very long, and twisted once and a half round its own axis, so that the ileum was forced up into the upper part of the abdomen, while the jejunum lay deeper under the ileum, the whole convolution of this intestine lying chiefly in the left umbilical region. Under the string of mesentery, and compressed by it against the left side of the vertebral column, the ileum, about a foot and a half from its junction with the cæcum, ran from above downwards and towards the right side, while the upper portion of the jejunum ran under the string in an opposite direction from below upwards and towards the left side. Consequently, the two terminating portions of the small intestine were constricted, and separated as it were by a ligature from the rest of the intestinal tube. At this point, the coats of the intestine were congested, of a dark red colour, soft, and friable.

CASE IV. A woman, aged forty-six, was admitted into the General Hospital on the 14th of January, 1833. Two years previously, she had been frequently attacked with pain in the back, and tympanitic swelling of the belly. About twelve months afterwards, she was seized, while lifting a load, with a sharp pain in the right inguinal region, and, from this period, was subject to tingling sensations in the feet, violent colic, and bilious vomiting. She stated on admission that her symptoms had been caused by an error in diet: there were, great anxiety and restlessness, tenderness of the abdomen, bilious vomiting, sinking of the pulse, and coldness of the extremities. She died on the fifteenth. In addition to remarkable degeneration of the right ovary, the following phenomena were observed. The colon and rectum were contracted, pale, and empty. The right ovary, which was enlarged so much as to measure nine inches in length, had ascended out of the pelvis and lay in the left iliac region. The lower end of the tumour was attached to the broad ligament of the uterus, the upper end was directed obliquely upwards and outwards, and attached by a membranous band about an inch in breadth to the upper surface of the mesentery of the jejunum. Under this enlarged ovary, ran, first the portion of intestine which lay behind its attachment to the mesentery, and lower down, another portion from the left to the right side; the intestine then descended into the right iliac fossa. At this place, the whole ileum with its mesentery, passing behind and beneath the cæcum, which had a loose mesentery, turned upwards and fell on the inner side of the ascending colon, and over the ovarian tumour. In this way the cæcum was at this point separated from the ascending colon, and strictured so as scarcely to admit the passage of a probe. It was greatly distended, and projected beyond the surrounding convolutions of the intestines, close to the ovarian tumour. Its coats, as well as those of the small intestine, were thickened and injected, and both intestines were loaded with fecal matter.

Case v. A woman, aged forty-eight, was brought into the General Hospital, in the latter end of May, 1834, with symptoms of strangulated hernia. The intestine was replaced with facility, and with some relief of her symptoms, but she died on the 2d of June. About two pints of a dirty greyish fluid, mixed with fecal matter, were found in the cavity of the abdomen. The small intestine was distended, and presented marks of congestion and peritoneal inflammation. One of the convolutions of the ileum adhered to the commencement of the hernial sac, which had escaped through the right inguinal canal, and, with its mesentery, formed a firm ridge or column directed from below upwards. The next portion of the ileum, which was about three feet in length, had passed under this column from within outwards, and then upwards and inwards, and at the same time was turned over it opposite the brim of the pelvis. In this way the canal of the ascending and descending portion of the gut was compressed against the column, and strictured. The strictured parts were of a dark red colour, and at one spot exhibited a perforation about the size of a pea. There was no appearance of stricture in the hernial sac.

Case vi. A man, aged sixty-one, was admitted into the General Hospital in April, 1830, with symptoms of inflammation of the bowels. He became gradually worse, had feculent vomiting, and died on the eighth day of his illness. He had laboured, during the latter part of his life, under obstinate costiveness, colic, and swelling of the abdomen, and was said to have brought on the last fatal attack by carrying a heavy load. Effusions of serum and lymph were found in the cavity of the abdomen; the small intestine, and the ascending and transverse colon, were greatly distended, their coats congested, discoloured, and friable; the descending colon and rectum were pale and contracted. The mesentery was very long, and exhibited several adhesions; the mesocæcum and mesocolon were also uncommonly long and lax. In consequence of the laxity of their attachments, the cæcum and colon, as far as the right flexure, had twisted on their axis from below and without upwards and inwards, and lay in the left side of the abdominal cavity. The mesentery of the small intestine, following this track, and running from below upwards, had thrown itself with its intestine across the transverse colon, and from thence descended into the right lumbar and iliac regions. In this way, the part at which the fold formed by the twisting of the intestine round its own axis was situated, was further strictured by the mesentery which lay across it, and compressed it still more tightly as the weight of its intestine increased. At the point of stricture, the intestine was friable and gangrenous, and exhibited perforations, through which the fluid fecal matter was dropping into the cavity of the abdomen.

Pure, uncomplicated cases of the second species of internal strangulation, occur, like those of the first species, only in persons advanced in life; when present at an earlier period, the tendency to their development is, in general, favoured by the existence of large or small hernia with adhesions of portions of the intestines to the walls of the abdomen, or by the pressure of accidental products, which alter the position and attachments of the abdominal viscera. They are observed most frequently in females, and in them, depend most probably on the local disposition of the parts contained in the abdomen. The rotation of a portion of intestine round its own axis can scarcely occur in any portion of the intestinal tube, except the ascending and transverse colon; rotation round the mesentery, as an axis, can happen only to the small intestine; whereas any portion of the small intestine, the cæcum, and, perhaps, also, the sigmoid flexure of the colon, may form the axis in the third variety. Strangulations of the second species are slow in their development, and appeared to be connected with the same external causes which determine those of the first species.

THIRD SPECIES. This is caused by some peculiar arrangement of parts, the result of original malformation, or of previous disease. *These strangulations of the intestine occur in circular or fissured spaces, formed, 1st, by fibres or bands of cellular membrane running from one organ to another; 2dly, by adhesion of the free end of the vermiform appendix to some spot of the walls of the abdomen, or to a portion of intestine or mesentery; 3dly, by adherent diverticula; 4thly, by the adhesion of two convolutions at a single point; 5thly, by perforations in the mesentery, or by fissures in an omentum altered by disease.* The following cases are given in illustration of this species of constriction.

CASE I. A woman, aged sixty-one, of full habit and robust constitution, was admitted into the General Hospital, on the 26th of June, 1828, with violent pain and distension of the abdomen, obstinate costiveness, fecal vomiting, sinking of the pulse, and coldness of the extremities. She died shortly after admission. On opening the abdomen, the following phenomena were observed: The whole of the intestines, with the exception of the descending colon, were distended, congested, and covered with patches of lymph. The cæcum, and ascending and transverse colon, were enlarged to four times their normal diameter; the coats of the intestine were thickened, vascular, and softened. From the transverse colon, close to its left flexure, a strong tendinous band ran obliquely along the left margin of the omentum, and, then running for the length of three inches towards the right side and downwards, attached itself to the right ovary, which projected into the upper pelvis. This band, making a turn and a half round the left flexure of the colon, its mesentery, and a portion of omentum, constricted the intestine at two points in such a manner as to obliterate its canal completely, so that air could not be forced through it. The descending colon and rectum were pale and contracted, the right ovary remarkably altered in its texture.

CASE II. Madame E., mother of several children, had suffered repeatedly from pain, tenderness, and distention of the abdomen, with great anxiety, and tendency to syncope. These symptoms were usually diminished on the occurrence of bilious vomiting, and completely disappeared after free evacuations from the bowels. In the Spring of 1832, she got a violent attack, which resisted every plan of treatment, and she died after thirty-six hours of intense suffering. In addition to inflammation of the peritoneum and effusion into the cavity of the abdomen, the following circumstances were noticed. Numerous threads and bands of cellular membrane extended from the uterus to the neighbouring viscera. One of these, about three quarters of an inch in breadth, stretched from the back of the uterus to the rectum, and, expanding in its course, attached itself to the latter down as far as the folds of Douglas. Between this band and the posterior wall of the uterus, there was a fissure, capable of admitting only a single finger, through which a loop of the ileum about a foot in length had passed, and was so much constricted, that a probe could scarcely be introduced between it and the margin of the fissure. The strangulated portion of intestine was distended with air and a fluid of a dark red colour, its coats measuring a line and a half in thickness, friable, and intensely congested.

CASE III. A servant, aged twenty-four, of powerful muscular make, who had frequently laboured under violent pain of the bowels, was admitted into the General Hospital in 1829, with symptoms of obstinate costiveness, followed by inflammation of the bowels and feculent vomiting, and died shortly after admission, notwithstanding the sedulous employment of every means likely to afford relief. On opening the abdomen, traces of extensive peritoneal inflammation were visible. The small intestine, to within about a foot and a half of its termination, was distended with air and feces to three times its normal diameter; the remainder, and the whole track of the large intestine, were contracted. At the point of separation between the distended and narrowed portion of the ileum, a diverticulum, five inches in length, and narrowed at certain points to the thickness of a piece of whipcord, descended from the upper wall of the intestine through a noose, (to be described presently,) and, about two inches from its attachment, adhered by firm cellular membrane to the under surface of the mesentery of a portion of intestine which ran parallel to it. Through the ring formed in this way, and which was about two inches and a half in diameter, passed, in the first place, the loop of intestine between the two fixed points of the diverticulum, and then a second and a third loop; the surface of these loops exhibited four spots excessively constricted and gangrenous, particularly that which lay immediately above or in front of the place where the diverticulum quitted the intestine.

Case iv. A man, aged thirty-six, was brought into the General Hospital, on the 3d of May, 1835, with the following symptoms: Violent pain and tension of the belly, tenderness on pressure, anxiety, thirst, vomiting, cool skin, small, frequent, wiry pulse. He attributed his illness to cold caught by sitting on the grass. His symptoms yielded to the usual remedies for some time, but returned with violence on the 8th of May, accompanied by obstinate costiveness and feculent vomiting, and he died on the 12th. On dissection, the following state of the intestinal tube was observed, in addition to extensive peritoneal inflammation. A ligamentous band of cellular membrane, two inches and a half in length, and half an inch in breadth, was stretched across the anterior surface of the mesentery of a loop of the ileum about a foot in length; the two ends of this band were inserted into the mesentery, close to the intestine, and were about three inches distant from each other at their termination. At the left side, close to the insertion of this ligament, the loop of intestine turned forwards and upwards over the ligament, and then descended through the fissure formed between it and the mesentery. In this way, the ligament was twisted into a dense string, about the thickness of a moderate packthread, and rendered half an inch shorter, so as to exert a strong degree of compression on the loop of intestine and its mesentery. The incarcerated portion of intestine was greatly thickened, infiltrated with blood, and of a dark green colour. The intestinal tube, above the stricture, was inflamed and distended; below it, pale and contracted.

Case v. A woman, aged thirty-one, of robust habit, was admitted into the General Hospital on the 21st of September, 1828, with symptoms of violent enteritis, obstinate costiveness, and vomiting, and died about twenty hours after her arrival. It was stated, that about three years previously, during her third pregnancy, she had felt an acute pain and sense of laceration in the umbilical region, while lifting a sack of meal. The pain yielded to rest, but returned whenever her bowels became confined, and was generally relieved by aperients. The peritoneum was extensively inflamed, the coats of the intestines discoloured, soft, and friable. A hole about three inches in diameter, and with a firm, rounded, smooth margin, was discovered in the mesentery of the ileum close to the cæcum, through which the cæcum had passed from behind forwards. In consequence of this, the ascending colon was half twisted round its own axis, and so firmly compressed against the margin of the perforation, that even air could not be forced through it. Below, the cæcum had drawn with it through the aperture a considerable portion of the ileum, which with its mesentery was twisted several times, where it lay next the lower half of the ring. The cæcum was swelled to the size of a large gourd, forced into the left iliac region, and surrounded by the folds of the distended ileum; beyond the constricted part, the large intestine was pale and contracted.

The following conclusions are deducible from the foregoing cases, with reference to strangulations of the third species. 1st. Cases of this description are very numerous; indeed, they appear from Professor Wagner's Essay to be the most frequent. 2d. They occur, with few exceptions, in youth and middle age, and particularly in females. 3d. They arise most probably from every cause capable of altering the position of certain portions of the intestine, and determining their passage through the ring-shaped or fissured openings. 4th. The tract of the small intestine is most liable to this species of strangulation; it may happen also to the freer and more moveable portions of the large intestine. 5th. The abdominal affections observed occasionally in certain individuals for many years before the fatal termination, authorize us to conclude that strangulation of this kind, with many of its varieties, may frequently take place, but that the intestine may be again released, and that rest, but still more, mild purgatives, by their effect on the vermicular motions of the intestines, may contribute largely to the production of this result.

From the consideration of all the foregoing cases Dr. Rokitansky draws the following inferences with reference to diagnosis:—

1. No age precludes the possibility of the occurrence of internal strangulations of the intestines; they occur, however, most frequently in the middle and advanced periods of life.

2. For a longer or shorter period before the fatal termination, the patient is attacked from time to time with symptoms indicating a strangulation of the intestine. These originate either from an error in diet, or from some violent bodily exertion, and generally commence with a sudden cutting pain in the bowels, (which, in some cases, proceeds from a determinate point,) followed by more or less rapid, visible distention of the abdomen, tympanitis, constriction of the chest, sense of anxiety, nausea and vomiting, according to the violence and duration of the strangulation. In addition to these affections, and even without them, the patients generally labour under a sluggish state of the bowels, and even costiveness, lasting for a considerable time. By rest, gentle aperients, and favorable positions of the body, these symptoms are frequently mitigated or dissipated, but are again reproduced by the original cause, and finally terminate in a fatal attack.

3. This attack generally arises from some error in diet, and sets in with *sudden* and violent pain in the bowels, which gradually extends over the whole abdomen. The belly is excessively distended and tympanitic, the respiration laboured, the eyes sunk, the countenance altered, and expressive of great anxiety. The alvine evacuations are small and scanty, or there is obstinate costiveness. The patient vomits, at first bilious, and afterwards feculent matter; the temperature sinks, and the pulse can scarcely be felt. A remission of the symptoms usually takes place shortly before death.

4. The course of this affection is in general very rapid; it seldom kills the patient before the second day, and frequently runs on for six, eight, or ten days. It rarely extends to the third week, and when it does, is interrupted by remissions and apparent improvements.

5. It may be distinguished, in most cases, by the appearance of the patient; by the preceding attacks, their origin from a determinate cause, and their course; by the intervals of ease between the attacks; by the suddenness of the attack, and its progressive increase after a certain period, and finally by the insurmountable costiveness.

With respect to treatment, Dr. Rokitansky rejects all medicine, but particularly purgatives, when the disease has reached a certain point, and proposes *the knife* as the only means of relief. He gives some general directions respecting the best mode of relieving the stricture, and preventing its return, and concludes by remarking, that further observations must decide on the admissibility of the plan of treatment which he has proposed.

*Medicinische Jahrbücher des Öst. St.* xix. Band. iv. Stück, 1836.

---

*On the Ciliary Motions in the Brain.* By M. PURKINGE.

IT has at length occurred to me to discover cilia, and their motions, in all the cerebral cavities of mammalia. In the preceding summer, while examining the *chordæ Bergmannicæ*, having observed, on fine slices of epithelium, a structure similar to that of the ciliary membranes, I conjectured that it would be found to possess the same function. Accordingly I made many investigations with the view of discovering this, but in vain, until the 28th of May of the present year (1836,) when I detected the ciliary motions, in the greatest activity, in the brain of the full-grown fœtus of a sheep, about thirty hours after death. The cilia were very distinct on all the walls of the cerebral cavities, and even where they were not in motion. I traced the motions, without difficulty, through the third ventricle into the infundibulum, then through the aqueduct of Silvius into the fourth ventricle. Here there was no motion perceptible, but the cilia themselves were distinctly visible, although somewhat shorter than in the former situations. The cilia are proportionately long pointed, (not ragged as in the bronchi,) and vibrate thong-shaped, (peitschenförmig:) we can also distinguish a layer of granules, in which they are fixed, and which can be easily stripped off without destroying the continuity of the epithelium. I recently observed them in the brain of a sheep; and Dr. Valentin saw them in the full-grown fœtus of a sow; but they could not be detected in a much younger fœtus of the same animal, probably because the parts are too fine for our gross instruments. In these observations I could remark that the cilia in the ventricles of the brain were much more sensitive and easily destroyed than in any other structure. I could not detect them in the brain of a sparrow, nor in a carp, nor in a diseased human brain. Probably they are in all parts very transient, yet readily reproducible; at least, this may be considered as made out in regard to the ovaria and mucous membrane of the nose.

*Müller's Archiv.* No. III. and IV. 1836.

---

*On the Ciliary Motions in Man.* By C. T. von Siebold.

The author of this paper has continued the experiments of Purkinge and Valentin, and he has added some facts which he has himself noticed, respecting the existence of the vibrating cilia upon the mucous membranes in man. He found these cilia upon the whole surface of a nasal polypus, one hour after its removal from an adult. The length of these ciliæ was 0.0028 and 0.0022 of an English line. The motions of the cilia upon one polypus which was examined, were found to be quite regular in their rhythm: in some parts they moved backwards and forwards 300 times, in others 320 times, in others 190 times, in a minute. The movements were always in the same direction; and, when once they ceased, were never resumed. By a very accurate examination, M. Siebold says that he has ascertained that each cilium curves its free extremity towards the part to which it moves, and that small globules of mucus, when in the vicinity of this oscillating body, are thus propelled in the same direction as the curve of the cilia. On condylomata at the entrance of the vagina no cilia could be discovered. They were also not found on the bronchial mucous membranes of those who had died of pneumonia, or of those who had suffered before death from a copious bronchial mucous secretion. In warm weather, also, it is useless to look in the human corpse for the cilia upon mucous membranes. M. Siebold has examined other membranes without finding any cilia; e. g. synovial membranes, sheaths of tendons, the internal surface of arteries and veins, that also of the vessels of the placenta, as well as serous membranes in animals the mucous membrane of whose bronchial apparatus presented an abundance of cilia. M. Siebold recommends all those who are anxious to examine this phenomenon to first experiment upon bivalves, (Unio pictorum, Anodonta anatina, Cyclas cornea;) for in them the cilia are most evident and their motions most easily recognized, abounding as they do upon their gills, tentacula, intestinal canal, &c. Having once witnessed the structure in these animals, it will be readily ascertained in man and other animals. *Medicinische Zeitung*, No. 28. 1836.

---

*On Secondary Abscess.* By Dr. H. Nasse, of Bonn.

The object of the distinguished author of this memoir, who has himself had abundant opportunity of investigating the pathology of Secondary Abscess, is to compare the result of his own observations and views with those already published by Rose, Dance, Cruveilhier, and others.

Under the term "*Secondary Abscess*" are now included, as is well known, such collections of pus as arise in consequence of external injury, whether from accident or from surgical operations; from the latter more especially when they refer to the *veins.* Such secondary formations in internal organs may, however, be equally the result of spontaneous suppuration in external parts, the more readily if the latter have its seat in the veins. Three stages may be distinguished in the formation of secondary abscess. The first is marked by infiltration of blood; the second stage, that of incipient suppuration, by the yellow colour and a lax state of the affected part which is inclosed within an indurated vascular web. In the last stage pus is found collected within cells, and ultimately within a cavity, whilst its cyst has become yet more condensed, its surface still remaining vascular. One peculiarity of the secondary affection is, that the infiltration, whether of blood or pus, is distinctly circumscribed. The pus is usually yellow and thick, at times (as Mr. Rose observes) not unlike tubercular matter. Towards the centre, the mass is fluid.

1. *In individual organs.* In the *Lungs,* we at first discover dark red and perfectly circumscribed patches, rather lighter in colour, though otherwise much resembling the morbid spots occasioned by pulmonary apoplexy. The next change is to a yellow colour, varying in shades, and ultimately modified by a pale reddish tint. As in grey hepatization, the parenchyma yields a small quantity of puriform matter on compression. The more yellow the colour, the more lax the substance becomes, until at length it may be pressed into a pulpy mass. A great degree of porosity is remarkable in the parenchyma thus morbidly changed, especially during the second stage, and its specific gravity is, upon the whole, less than in common hepatization. As the suppurating process advances, the pus collects in numerous cells, whose envelopes consist of cellular tissue and blood-vessels. These envelopes first disappear at the centre and progressively towards the circumference, until the pus is at length contained in a single cavity with hard, dense, indurated, and vascular parietes. The pus now assumes a greenish yellow colour, is viscid, and contains membranous filaments, as appears on its being mixed with water. Both the number and the size of these pulmonary abscesses are extremely various, and in the same lung several abscesses are sometimes discovered in various stages of progress. Their seat is commonly in the inferior lobes, and at the then lower extremity of the latter; they are, however, occasionally met with in the upper lobes as well. The posterior portion of the lungs appears most liable to them. Rose asserts that the greater number of abscesses are seated directly beneath the pleura; but they are frequently observed in the very midst of the viscus. When at all superficial, they may be recognized from without the lung, by a corresponding yellowish spot on its surface. The observation of Cruveilhier is deserving of great attention, that the ramifications of the pulmonary artery leading to the morbid part are in some cases found to contain coagula extending to the minute extremities of the vessels. In the smallest branches these coagula are blood-coloured and tolerably firm; in the larger branches yellow and soft. Some of them contain puriform matter within their disorganized mass. In several cases examined by Dr. Nasse, some of the branches of the pulmonary artery contained perfectly formed pus; and, on following the course of the artery, he discovered decided traces of inflammation in its coats, accompanied by a species of gelatinous exudation. Thus the suppurative process appeared to be further advanced in the smaller branches than in the larger and more conspicuously inflamed ones. When the lungs contain these abscesses, the pleura almost always exhibits inflammation, is usually enveloped in a false membrane, and sometimes contains within its cavity a reddish, fœtid, and flocculent, or even purulent fluid. If this fluid be at all copious, the lung is necessarily compressed by it, and yet it will be found to contain more or fewer abscesses; a proof that the formation of the latter preceded the other morbid phenomenon.

2. *In the Liver.* At first dark brown spots are discovered, which Cruveilhier ascribes to a discoloration of the granules of the parenchyma. At a later period, when purulent infiltration commences, these granules become yellow, and are surrounded by brownish slate-coloured laminæ, so that the whole part assumes the appearance of granite. As suppuration proceeds still farther, the colours gradually blend together, until the whole presents a homogeneous mass. The size of the abscesses varies from that of a pea to that of a hen's egg; their number from a single one to thirty or forty, but they are more frequently numerous than otherwise. When they occur near to the surface, the peritoneal covering of the liver assumes, close above their seat, a slaty, but in the more developed stage, a yellow colour. The form of the abscesses is irregular, round or oval, and indented. The mass they contain is yellow, gritty, except in colour, not unlike macerated parenchyma of the liver. It adheres to the parietes of the abscess, but is easily separated from it by pressure. The author never found *healthy* pus, or, like Cruveilhier, matter resembling turbid milk, in those cavities the parietes of which constitute a perfect cyst. The hepatic veins sometimes penetrate the abscess without appearing injured. Several small veins (probably belonging to the vena portæ,) leading away from the cysts, were in certain instances found to contain purulent matter; whilst the larger and rather more remote veins exhibited coagula. In one case of some standing, the branches of the vena portæ leading to an abscess were found to have their channels obliterated. In the vicinity of large abscesses yellow spots were here and there observed.

3. In the *Spleen.* According to Cruveilhier the spots are at first dark red, and of a spherical form, precisely as in apoplexy. Hennin states that suppuration is preceded by the formation of indurated spots.

*The synovial membrane* was once found by the author in contact with healthy pus. The surface of the membrane exhibited roughness, but no inflammatory redness.

It is of the greatest importance to the pathology of this disease to determine, as far as possible, the local relations of secondary abscess to the primary affection. It is remarkable that French pathologists alone have persevered in affirming that injuries of the head are particularly disposed to affect the *liver* with secondary abscess. English pathologists, generally speaking, deny the truth of this proposition, and those of all other countries do not, upon the whole, ascribe any precedence of sympathy in this respect to the liver when compared with other organs, such as the lungs and pleura, the spleen and peritoneum. The results of Dr. Nasse's observations would seem to prove that injuries of the head are most frequently attended by secondary affections of the lungs, together with the pleura, or of the lungs exclusively, and at times simply of the pleura. With the liver, the lungs are in most cases simultaneously attacked, or else the spleen. This latter organ is more commonly found filled with incipient or with perfect abscesses, in injuries of the head, than in those of any other part. Femoral wounds, amputations and fractures of the bones, far more easily occasion abscesses in the lungs only (with or without pleural exudation,) than in the liver alone, or in the lungs and liver at once. Abscesses in the lungs are as 11 to 3, when compared with those of the liver, and with those of both organs simultaneously, as 10 to 4. In femoral injuries, Dr. N. has never observed abscesses exclusively in the liver. According to Cruveilhier's experiments, mechanical irritation of the inferior vena cava in animals is always productive of pulmonary abscess. Pus has been detected within the hip-joint, and the bursa mucosa lately described by Fricke has been found replete with it. The knee-joint has sometimes presented the same phenomenon, as well as most of the other joints. In one case of suppuration at the shoulder-joint, the pus had burst through the capsular ligament and had flowed down the arm, filling the cellular sheaths of the veins, so as at first to make it appear as if these vessels themselves were charged with pus. In like manner pus has been discovered collected within muscles.

Secondary inflammation less rarely attacks the intestinal mucous membrane than either the peritonæum or the pericardium. In an inflammation of the great saphena vein, Laver found the pleura to be the exclusive seat of the secondary affection. Others have observed phrenitis, though unattended with suppuration, to succeed idiopathic phlebitis. According to Dr. N.'s own experience, amputation is more frequently productive of abscess in the lungs than in any other organ. From injury of the upper extremities, he more commonly met with hepatic abscess, alone or conjoined with that of the lungs. Dr. Nasse's experiments, in which he injected from two to three drachms of ill-conditioned pus into the jugular vein of dogs, had no apparent effect upon the animals. Nor did he, on killing them, after the lapse of a few days, discover any morbid symptom in any of their internal organs. With other physiologists the same kind of injections, repeated until they caused the animal's death, were found to have produced signs of inflammation in the heart, the lungs, and the intestines.

Suppuration having its seat in the veins which proceed from the uterus, the bladder, or the rectum, may occasion secondary abscess in the liver, without sympathetic affection of the lungs. The latter are however occasionally attacked from the same cause without any concomitant affection of the liver. It is singular that whilst one author refers pulmonary, hepatic, splenitic, and cerebral disease to suppuration of the uterine veins, Dance, who had equal opportunity for observation in this respect, excludes both the liver and the brain from the list of organs thus affected and substitutes for them the synovial membranes.

The symptoms connected with the incipient stage of secondary inflammation always indicate a violent affection of the whole system. They seldom begin to develope themselves before the tenth day from the commencement of the primary affection, most frequently at some period between the tenth and the twentieth day, and occasionally not until after the lapse of several weeks (Cruveilhier), or even a couple of months, in rare instances. We should, in fact, scarcely overstep the truth in affirming that the generation of secondary abscess continues to be a possible event so long as the act of primary suppuration lasts, more especially where bones are laid bare and imbedded in pus. No period of age, no constitution, even the most robust, is exempt from the danger, although those addicted to the use of ardent spirits appear most of all exposed to it. The treatment of the primary disorder seems not in any sensible degree to influence the development of the deuteropathic affection. In every case the attack sets in with a severe affection of the vascular system; the patient is at first almost always seized with rigors, which, without being followed by any considerable heat, end in copious perspiration. The rigors commonly return several times, either at irregular periods, or, as is sometimes the case, as regularly as in quotidian fever.

Although the great branches and the trunks of the veins of a member may be discovered in a state of suppuration *after death*, the development of the more general symptoms is nevertheless seldom preceded by the phenomena of acute phlebitis. A short time previously, the wound may appear inflamed, or the limb exhibit an œdematous swelling, from which latter circumstance a disturbed state of the venous circulation may with certainty be inferred. After the attack of rigors, the suppurating surface *generally* dries up and presents a pale and deadened appearance, remaining thus or secreting an ichorous fluid during the whole of the ensuing period. Occasionally, however, suppuration continues unaltered, both in quantity and in quality; nor, if the injured part be inflamed, is the inflammation always and necessarily arrested by the secondary affection. The commencement of the constitutional symptoms is in most instances very sudden, the patient having up to that moment felt tolerably well and comfortable. A more gradual development is of course observed when the locality of the primary lesion is in itself sufficient to occasion constitutional or sympathetic disturbance; as, for instance, in the vicinity of the brain. The secondary affection is at times unattended by any local symptoms during life; in more instances, however, such symptoms display themselves sooner or later, but most commonly within a couple of days after the constitutional ones have set in. Before this occurs, the primary suppuration, which may have either become deteriorated or been suppressed, is sometimes found to resume its healthy character. Where the lungs or merely their serous envelope are affected, cough, oppression of the chest, dyspnœa, and anxiety mostly ensue. Percussion and auscultation throw but little light upon the nature of the existing affection, and it is only a copious discharge into the pleural cavity that can be recognized by such means: examples were met with by Dr. N., wherein none of the symptoms above adverted to manifested themselves. Thus, a youth, aged fifteen, although continually uttering plaintive cries, had no cough, and complained of nothing particular. On opening the body, an extraordinary quantity of a puriform fluid was discovered within the pleural sacs, besides several extensive abscesses in the lungs. In another instance the existence of lobular pneumonia remained undetected during life; this however arose from a complication with disease of the brain, at the convex portion of which large abscesses had formed. Where the seat of secondary disorder is the liver, the patients generally complain of pain in the left hypochondrium (which however does not always increase on pressure,) as well as of pain near the right shoulder-blade. Jaundice is a very frequent, though by no means a necessary attendant upon this hepatic affection.

During the development of the local symptoms, the patient sinks into a typhous (adynamic) state, which, if it comes on at an early period, may suppress all expression of pain. The tongue becomes dry, oftentimes brown, the skin hot; the strength rapidly sinks; the patient evinces great restlessness, is usually delirious, often continuedly so, though sometimes only during the periods of febrile exacerbation; ultimately he falls into a stupor, and it is only in the more rare instances that the brain maintains its activity unimpaired until death.

The pulse is in general very frequent, (though, in rare cases, slow;) often irregular, and weak. The fever is of the continued kind, occasionally with exacerbations. When the disease has assumed the course above described, it is certain to end in death, frequently within a few days (from three to five,) from the commencement of the secondary symptoms, or even still more speedily; in not a few instances very suddenly and unexpectedly.

The symptoms which succeed the injection of pus into the veins of animals differ from those observed in the human subjects, and that no less in the cases where the animals continue to live, than in those where the artificial disease occasions their death. It is worthy of remark that the system here strives to disburden itself of the alien matter by means of augmented excretions, both of fæces and urine.

[The observations and notices, of which the foregoing is rather an abstract than a translation, constitute the basis of a long and able enquiry into the pathogenesis of secondary abscesses. These the author appears inclined to consider as connected with phlebitis, which in its turn is supposed to originate in deterioration of the blood, owing to the reabsorption of pus or other disorganized matter into the venous circulation. Our limits prevent us from following the author through this interesting enquiry, for the details of which we must refer to the original article.]

*Rust's Magazin. Fünfundvierzigsten Bandes, Drittes Heft.*

---

*Observations on the best Mode of Demonstrating the Internal Structure of the Heart, and on the Septum of the Auricles in Man.* By Professor Retzius.

With a view to obtain a more instructive representation of the heart in its natural state than has hitherto been done, the author has for some time pursued the following method, which exhibits the cavities and the situation of the valves in a distinct and correct manner. The heart having been removed from the subject, in connexion with the liver, the venæ cavæ, the aorta, and the lungs, and having been properly freed from blood by injections of water, is steeped in a mixture of oil of turpentine and spirits of wine. The cavities are then amply injected through the pulmonary and superior venæ cavæ, the aorta, and the pulmonary artery, with a mixture of white wax and oil of turpentine. As soon as the mass is firm, the heart, together with the great vessels, is separated from the rest, the extremities of the vessels are tied, and the preparation, having been cleaned with the scalpel, is left to dry. When thoroughly dried, it is macerated in spirits of turpentine, till the wax is softened or entirely dissolved. In this manner the heart and arteries are emptied after the walls have dried over the wax forms, which were true casts of the natural cavities, their septa and valves, &c. The parietes themselves, being impregnated with turpentine, lose little of their natural thickness. The parietes may now be either cut open or rendered transparent with the aid of resins, so that the internal structure can be examined. Another method of preparation, somewhat less advantageous, is to open the auricles, ventricles, and blood-vessels, then fill them up with cotton, or to leave them unopened, but to fill them with proof spirit, afterwards suspending them in the same medium. The water contained in the heart's tissue is attracted by the spirit; the walls thus become stiffened, and retain their form even after the alcohol has been removed. Preparations of this kind are to be met with in Hunter's museum.

On examining a heart thus prepared, the first observation we make is that the left auricle forms an oblong pouch, having almost a horizontal position, its right extremity encroaching upon the domain of the right auricle, the situation of which is nearly vertical. At the point where the two pouches meet, the septum of the auricles developes itself with its inferior portion almost lying across the mouth of the vena cava. This is the exact spot which in the fœtus is occupied by the foramen ovale and its border, and the partition itself consists of the thickened valve, by the adhesion of which with the neighbouring parts the foramen ovale was closed up. The upper part of the septum forms the imperfect septum auricularum in the fœtus. If the right auricle be opened, the left being filled up, we discover a protuberance presented by the upper part of the border of the obliterated valve. This, together with the convexity formed above it by the septum, probably constitutes the tubercle mentioned by Lower.

The arrangement of the septum in its different parts has the greatest influence over the functions of the heart under the various circumstances of life. If the body be at rest, the blood flows gently from the lower parts into the right auricle, but if it be pumped up by hard breathing, as in those affected with dyspnœa, the influx becomes far more hurried. It is still more accelerated by an uninterrupted exercise of the muscles of the lower extremities, as the muscles then press on the parietes of the large veins, and thus force the blood onwards towards the auricle, whilst its retrogression is prevented by the valves. The curvature of the septum, the tendency of the circular muscles around the foramen ovale, and the curvatures at the entrance of the venæ cavæ, prevent the blood from penetrating from the inferior into the superior venæ cavæ, or vice versâ. If it were otherwise, apoplexy would easily occur in the former instance, and injurious effects on the liver in the second. *Kgl. Wetensk. Acad. Handlinger.* 1835.

